# Ln_2_F_2_(OH_2_)(MoO_3_)_2_(SeO_3_)_2_: Promising Multifunctional Nonlinear Optical Materials Created by Partial Fluorination Strategy under Corrosion Resistant Supercritical Reactions

**DOI:** 10.1002/advs.202304463

**Published:** 2023-10-23

**Authors:** Yun‐Xiang Ma, Peng‐Fei Li, Chun‐Li Hu, Jiang‐Gao Mao, Fang Kong

**Affiliations:** ^1^ State Key Laboratory of Structural Chemistry Fujian Institute of Research on the Structure of Matter Chinese Academy of Sciences Fuzhou 350002 P. R. China; ^2^ University of Chinese Academy of Sciences Beijing 100049 P. R. China

**Keywords:** multifunctional materials, nonlinear optical crystals, partial fluorination, second harmonic generation, supercritical hydrothermal method

## Abstract

It has historically been exceedingly challenging to create physically and chemically stable lanthanide compounds with strong second harmonic generation (SHG) due to their strong preference to central symmetry. In this work, five new non‐centrosymmetric lanthanide selenites, namely, Ln_2_F_2_(OH_2_)(MoO_3_)_2_(SeO_3_)_2_ (Ln = Sm, Eu, Gd, Tb and Dy), are achieved by partial fluorination of the lanthanide oxygen polyhedron. An HF corrosion resistant supercritical hydrothermal method is developed, which is a facile and universal method for HF corrosion and high‐temperature high‐pressure environment. The title compounds displayed a novel 3D framework composed of 1D molybdenum selenite chains bridged by Ln_2_F_2_O_12_(OH_2_) dimers. Their powder SHG responses showed a large difference, ranging from 1.0 to 9.0 × KH_2_PO_4_ (KDP) at 1064 nm. The half‐filled Gd compound exhibited very strong SHG efficiency of up to 1.2 × KTP (KTiOPO_4_) at 2050 nm. Compounds Tb and Gd are the first lanthanide selenites with SHG intensity reaching KTP level, which is very rare in this system. Furthermore, these compounds can also possess excellent physicochemical stability and strong luminescence emission, indicating that they are promising multifunctional nonlinear optical materials. This work offered an effective way for design and synthesis of multifunctional and high‐performant nonlinear optical materials.

## Graphical abstract

A general corrosion resistant supercritical reaction method was proposed. By partial fluorination strategy, new and polar rare earth selenites are first achieved in Ln‐Mo‐F‐SeO_3_ system. They can display strong SHG and luminescent responses simultaneously. Specifically, Gd_2_F_2_(OH_2_)(MoO_3_)_2_(SeO_3_)_2_ features a large and phase matchable SHG intensity of 1.2 × KTP at 2050 nm.

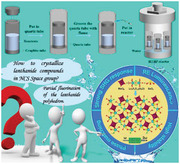

## 1 Introduction

Nonlinear optical (NLO) crystals, such as second‐harmonic generation (SHG) materials, are one of the key components of laser technology due to their irreplaceable role in expanding the available laser wavelengths.^[^ ^1^, ^2^, ^3^, ^4^, ^5^, ^6^, ^7^ ^]^ After more than half a century of development, many high‐performance SHG materials have been synthesized.^[^ ^8^, ^9^, ^10^ ^]^ As modern photonic technology continues to advance in miniaturization and integration, there is a growing demand for multifunctional nonlinear optical materials.^[^ ^11^ ^]^ Lanthanide elements are the treasure house of multifunctional materials with excellent magnetic, optical, and electrical properties.^[^ ^12^, ^13^, ^14^, ^15^ ^]^ However, new non‐centrosymmetric (NCS) lanthanide compounds are hard to obtain because the high coordinated lanthanide polyhedrons are preferred to form CS infinite frameworks. As the retrieved result from the Inorganic Crystal Structure Database, the proportion of NCS compounds in lanthanide oxides is ≈12.4%, which is far lower than that in all inorganic compounds (≈18%). As we know, NCS structure is the prerequisite for SHG crystals.^[^ ^16^ ^]^

It is reported that the following cations or groups are beneficial to the formation of NCS structures: i) lone pair cations (Se^4+^, Te^4+^, I^5+^, ect.),^[^ ^16^, ^17^, ^18^ ^]^ ii) d^0^ transition metal (TM) centered octahedra (MoO_6_, WO_6_, VO_6_, etc.),^[^ ^19^ ^]^ iii) π‐conjugated groups (BO_3_, NO_3_, CO_3_, ect.),^[^ ^20^, ^21^ ^]^ iv) tetrahedra groups (PO_4_, BO_4_, GaS_4_, etc.),^[^ ^22^, ^23^, ^24^ ^]^ v) d^10^ transition metals (Zn^2+^, Cd^2+^, Hg^2+^).^[^ ^25^ ^]^ Self‐assembly of the above ions or groups is an effective way to design and synthesize new SHG materials. For example, the first antimony(III) borate SbB_3_O_6_ was formed by SbO_4_, BO_4,_ and BO_3_ groups, exhibiting a strong SHG intensity of ≈3.5 × KDP,^[^ ^26^ ^]^ Furthermore, fluorine element with the most electronegativity can improve the comprehensive properties of SHG materials.^[^ ^27^, ^28^ ^]^ Prof. Pan Shilie's group reported a series of NCS fluorooxoborates or borate fluorides that are expected to be the next generation of deep UV NLO materials by introducing fluorine element into classic borate system, such as NH_4_B_4_O_6_F (cutoff edge: 156 nm, SHG response: 3 × KDP)^[^ ^29^ ^]^ and Sr_3_B_6_O_11_F_2_ (cutoff edge: <190 nm, SHG response: 2.5 × KDP).^[^ ^30^ ^]^ etc.

Based on the above considerations, we intended to introduce the d^0^ TM of Mo(VI) with the largest distortion degree and the asymmetric selenite groups with lone pair cations into lanthanide oxide system to explore new multifunctional NLO materials. However, the reported lanthanide molybdenum selenium (IV) oxides were all crystalized in CS space group and SHG‐inactive.^[^ ^31^, ^32^, ^33^ ^]^ The nonpolar lanthanide polyhedrons in these compounds were interconnected into CS infinite expanded structures, which hindered the effects of the polar groups.

If the infinite lanthanide network was depolymerized into separate units, the polar groups may play a major role in the structural symmetry, resulting the lattice transformation from CS to NCS. To reduce the interaction of the nonpolar units, partial fluorination of the lanthanide polyhedrons strategy was proposed due to the low valence state and weak bridging ability of fluorine ions. Through extensive literature research, we found that no compounds have been reported in Ln‐Mo‐F‐SeO_3_ system.

**Figure 1:**
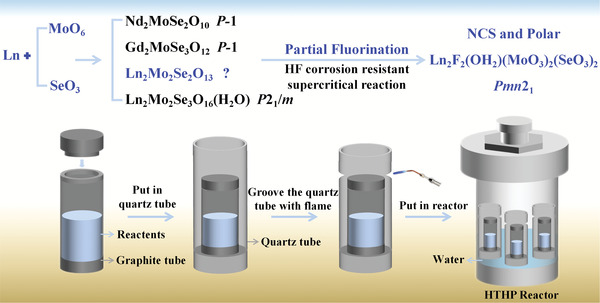
Schematic diagram of the strategy for design and synthesis of the title compounds.

Based on the known lanthanide molybdenum selenites, the molar ratio of Ln/Mo/Se was fixed on unreported 2/2/2 (**Figure** **1**).^[^ ^31^, ^34^ ^]^ To achieve the partial fluorination of lanthanide polyhedron, we have tried some regular synthesis methods, like medium/low‐temperature hydrothermal reactions and high‐temperature solid‐state reactions, but all failed. It is reported that the supercritical hydrothermal method is a distinctive synthesis technique that harnesses the exceptional properties of water in its supercritical state to facilitate reactions under conditions of elevated temperature and pressure.^[^ ^35^, ^36^, ^37^ ^]^ This approach holds promise for the discovery of novel NCS compounds. However, the reported supercritical hydrothermal methods cannot handle the reaction environments with both strong acids and fluoride ions. To settle this problem, we developed an acid and HF corrosion resistant supercritical hydrothermal method and achieved five new NSC lanthanide selenites, namely, Ln_2_F_2_(OH_2_)(MoO_3_)_2_(SeO_3_)_2_ (Ln = Sm, Eu, Gd, Tb and Dy) (**Table** **1**). They are promising multifunctional materials with strong SHG response and fluorescence performance. Herein, we report the syntheses, structures, and optical properties of the first NCS lanthanide molybdenum selenites.

**Table 1:** Crystal data and structural refinements for Ln_2_F_2_(OH_2_)(MoO_3_)_2_(SeO_3_)_2_ (Ln = Sm, Eu, Gd, Tb, and Dy).

Formula	Sm_2_F_2_(OH_2_)(MoO_3_)_2_ (SeO_3_)_2_	Eu_2_F_2_(OH_2_)(MoO_3_)_2_ (SeO_3_)_2_	Gd_2_F_2_(OH_2_)(MoO_3_)_2_ (SeO_3_)_2_	Tb_2_F_2_(OH_2_)(MoO_3_)_2_ (SeO_3_)_2_	Dy_2_F_2_(OH_2_)(MoO_3_)_2_ (SeO_3_)_2_
CCDC No.	2213 643	2213 644	2213 645	2213 646	2213 647
fw	898.52	901.74	912.32	915.66	922.82
Space group	*Pmn*2_1_	*Pmn*2_1_	*Pmn*2_1_	*Pmn*2_1_	*Pmn*2_1_
a (Å)	7.0766(6)	7.0775(6)	7.0624(5)	7.0633(2)	7.0329(7)
b (Å)	9.1608(8)	9.1439(8)	9.1047(7)	9.0767(3)	9.0184(10)
c (Å)	9.0309(7)	9.0172(7)	8.9963(7)	8.9761(3)	8.9468(9)
V (Å^3^)	585.45(8)	583.56(8)	578.47(8)	575.47(3)	567.46(10)
Temperature	293 K	293 K	293 K	293 K	293 K
Z	2	2	2	2	2
Dc (g cm^−3^)	5.097	5.132	5.238	5.284	5.401
µ(MoKα) (mm^−1^)	18.293	19.037	19.827	20.695	21.692
GOF on F^2^	1.028	1.069	1.092	1.048	1.031
Flack factor	−0.018(19)	−0.002(19)	0.01(3)	0.008(17)	0.00(2)
R_1_, wR_2_ [I>2σ(I)]^a)^	0.0285, 0.0612	0.0291, 0.0575	0.0415, 0.0917	0.0275, 0.0560	0.0374, 0.0788
R_1_,wR_2_ (all data)	0.0296, 0.0620	0.0316, 0.0597	0.0431, 0.0928	0.0295, 0.0573	0.0414, 0.0813

^a)^ R_1_ = ∑||Fo| − |Fc||/∑|Fo|, wR_2_ = {∑w[(Fo)^2^ − (Fc)^2^]^2^/∑w[(Fo)^2^]^2^}R^1/2^.

## 2 Results and Discussion

The single crystals of the title compounds were synthesized by lanthanide oxide, MoO_3_, SeO_2,_ and hydrofluoric acid under supercritical hydrothermal conditions (Figure 1). The reaction temperature and pressure were 380±1 °C and ≈23 Mpa, respectively. The fluorine source of HF is very important in promoting the crystallization of the title compounds. If the less corrosive fluorides, such as NaF or LnF_3_, were used, no crystals could be obtained. To prevent the corrosion of quartz tubes by hydrofluoric acid, the reactants were put in a graphite tube with cover, which was then placed in a slightly larger quartz tube. Use flame to groove the upper end of the quartz tube to prevent the cover of the graphite tube from spraying out. Such acid and HF corrosion resistant supercritical hydrothermal method resulted the first polar lanthanide d^0^‐TM selenite compounds of Ln_2_F_2_(OH_2_)(MoO_3_)_2_(SeO_3_)_2_ (Ln = Sm‐Dy) (Figures S1 and S2, Supporting Information).

**Figure 2:**
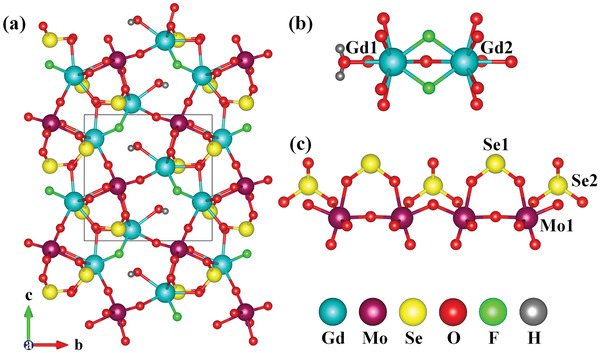
View of the 3D structure of Gd_2_F_2_(OH_2_)(MoO_3_)_2_(SeO_3_)_2_ along the a‐axis (a), the Gd_2_F2O_12_(H_2_O) dimer formed by Gd(1) and Gd(2) polyhedra (b), the 1D molybdenum selenite chain composed of MoO_6_ and SeO_3_ groups (c).

The isomorphic structures feature a novel 3D framework composed of 1D molybdenum selenite chains bridged by Ln_2_F_2_O_12_(H_2_O) clusters (**Figure** **2**a), which will be illustrated in detail, represented by the Gd compound. The asymmetric unit of Gd_2_F_2_(OH_2_)(MoO_3_)_2_(SeO_3_)_2_ contains two Gd, one Mo, two Se, eight O, and one F atoms, as well as one H_2_O molecule. The Gd atoms are nine‐coordinated with seven oxygen and two fluorine atoms. The Gd─O and Gd─F bond distances are in the ranges of 2.368(15)–2.511(15) Å and 2.317(9)–2.336(9) Å (Table S1, Supporting Information), respectively, which are close to those reported in related compounds.^[^ ^38^ ^]^ The Mo(1) atom is six‐coordinated with six oxygen atoms in a distorted octahedron, with the Mo─O bond distances falling in the range of 1.715(11)–2.219(9) Å. The Se(1) and Se(2) cations are in the center of ψ‐SeO_3_ tetrahedron with one vertex occupied by the lone pair electrons. The Se─O bond distances range from 1.642(14) to 1.756(10) Å. Bond valence calculations gave values of 3.27, 3.16, 6.06, 3.90, and 3.82 for Gd(1), Gd(2), Mo(1), Se(1), and Se(2), respectively (Table S2, Supporting Information), indicating that they are in the oxidation states of +3, +3, +6, +4 and +4, respectively.

Due to the partial fluorination of lanthanide polyhedrons, the connection between lanthanide polyhedra was restricted, which weakened the influence of lanthanide ions on the structural symmetry. Different from the infinite periodic structures formed by lanthanide oxide polyhedra in literatures, the Gd(1) and Gd(2) atoms in Gd_2_F_2_(OH_2_)(MoO_3_)_2_(SeO_3_)_2_ are face‐shared into a Gd_2_F_2_O_12_(H_2_O) dimer by two F^−^ and one O^2−^ ions (Figure 2b). The distorted MoO_6_ octahedra were corner‐shared into a molybdenum oxide chain with the SeO_3_ groups bridged on it, forming a new molybdenum selenite chain. As shown in Figure 2c, the selenite groups were arranged on the same side of the molybdenum oxide chain. Due to the blocking effect of the asymmetric SeO_3_ units, the MoO_6_ octahedron was distorted to the edge formed by O(5) and O(6) atoms, which were located away from the “blocking groups” and coordinated with lanthanide cations. The distorted degree of the MoO_6_ octahedron was calculated to be 1.0, which belongs to strong distortion. The molybdenum selenite chains were bridged by the Gd_2_F_2_O_12_(H_2_O) dimers into a 3D framework with pentagonal tunnels along the a‐axis. The coordinated water molecules were located at the odd member ring tunnels.

FTIR spectra for Ln_2_F_2_(OH_2_)(MoO_3_)_2_(SeO_3_)_2_ (Ln = Sm‐Dy) were recorded in the wave number range of 4000–400 cm^−1^ at room temperature (Figure S3, Supporting Information). It is noteworthy that these materials were barely absorbed in area of 1638–3396 cm^−1^. Peaks ≈1633–1638 cm^−1^ and 3396–3410 cm^−1^ can be attributed to the stretching vibration of O─H and the bending mode of H─O─H. As illustrated in the enlarged illustration, the bands associated with Se─O and Se─O─Se vibrations appear at 500–800 cm^−1^. The bands occurring at 850–950 cm^−1^ can be assigned to Mo─O and O─M─O vibrations.

UV–vis–NIR diffuse reflectance spectra studies indicate that the optical band gaps of Ln_2_F_2_(OH_2_)(MoO_3_)_2_(SeO_3_)_2_ are 3.20, 3.18, 3.15, 3.05, and 3.24 eV for Sm, Eu, Gd, Tb and Dy compounds respectively (Figure S4, Supporting Information). Sm_2_F_2_(MoO_3_)_2_(SeO_3_)_2_(H_2_O) is almost transparent in the range of 2500–1700 nm, and eight continuous small absorption peaks appear after 1700 nm at about 1593, 1552, 1516, 1407, 1375, 1255, 1227, and 1091 nm. Eu_2_F_2_(MoO_3_)_2_(SeO_3_)_2_(H_2_O) exhibits several distinct absorptions at 2110, 2035, and 1867 nm, and a wide absorption range at 910–581 nm. Gd_2_F_2_(OH_2_)(MoO_3_)_2_(SeO_3_)_2_ is almost transparent in the range of 2500–800 nm, except for a small peak ≈1969 nm. The Tb compound gave three small peaks at 1948, 1885, and 1787 nm, respectively. Dy_2_F_2_(MoO_3_)_2_(SeO_3_)_2_(H_2_O) gave ten peaks in the range from 1967 to 745 nm, corresponding to 1967, 1705, 1619, 1292, 1102, 909, 811, 794, 758, and 745 nm, respectively. These absorptions can be ascribed to the f‐f or f‐d transitions of the lanthanide cations.

**Figure 3:**
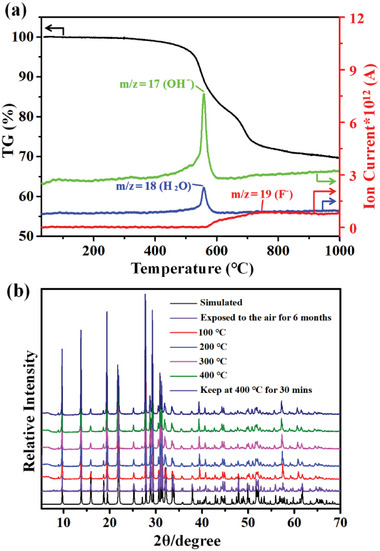
Thermogravimetry mass spectrometry of Gd_2_F_2_(OH_2_)(MoO_3_)_2_(SeO_3_)_2_ (a) and PXRD of Gd_2_F_2_(OH_2_)(MoO_3_)_2_(SeO_3_)_2_ at different conditions (b).

The thermal behaviors of Ln_2_F_2_(OH_2_)(MoO_3_)_2_(SeO_3_)_2_ (Ln = Sm‐Dy) were characterized by thermogravimetric analysis (TGA) (Figure S5, Supporting Information). They displayed similar decomposition curves corresponding to the loss of H_2_O, SeO_2_, and F_2_ molecules from 400 to 1000 °C. From the insert table of Figure S5 (Supporting Information) we can find that these compounds underwent incomplete weight loss at 1000 °C except for Gd_2_F_2_(OH_2_)(MoO_3_)_2_(SeO_3_)_2_. To disclose the decomposition temperature of F_2_ and H_2_O molecules from Gd_2_F_2_(OH_2_)(MoO_3_)_2_(SeO_3_)_2_, thermogravimetry mass spectrometry was performed (**Figure** **3**a). The results showed that the release of H_2_O started at about 400 °C and ended at about 600 °C while the loss of F element occurred at about 565 °C and continued to 1000 °C. Although these compounds contain coordination water, they can still be stabilized up to 400 °C, which was proved by the variable temperature X‐ray powder diffractions (Figure 3b). Such stability can be comparable to some of the anhydrous selenite SHG materials with halogen ions (Table S3, Supporting Information). Moreover, Gd_2_F_2_(OH_2_)(MoO_3_)_2_(SeO_3_)_2_ can also exhibit excellent air stability, as evidenced by its ability to maintain a well‐preserved crystal morphology even after six months, as confirmed by PXRD analysis (Figure 3b).

**Figure 4:**
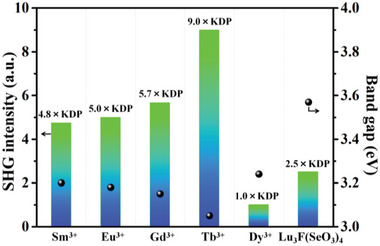
SHG intensity and band gap of Ln_2_F_2_(OH_2_)(MoO_3_)_2_(SeO_3_)_2_ (Ln = Sm‐Dy) compared with the reported Lu_3_F(SeO_3_)_4_.^[^ ^15^ ^]^

Powder SHG measurements revealed that the samples of Ln_2_F_2_(OH_2_)(MoO_3_)_2_(SeO_3_)_2_(Ln = Sm‐Dy) can display strong SHG signals of about 4.8, 5.0, 5.7, 9.0, and 1.0 × KDP at 1064 nm, respectively, which is much larger than that of Lu_3_F(SeO_3_)_4_ with the largest SHG intensity in reported lanthanide selenites (**Figure** **4**). We can find that their SHG intensities are totally different although they are isostructural. It is worth mentioning that the SHG intensities of these compounds have negative correlation with their optical band gaps, with the larger bandgap, the weaker the SHG intensity, which is consistent with the literatures.^[^ ^39^ ^]^ However, the largest difference of the bandgap is only 0.19 eV, but the SHG intensity is nine times different. We think such a difference should be caused by the different absorption around the wavelengths of incident and frequency doubling light. Due to the strong f‐f transitions of Dy(III), Dy_2_F_2_(MoO_3_)_2_(SeO_3_)_2_(H_2_O) displays the weakest SHG intensity in the five compounds. As to compounds Sm to Tb, their SHG intensity is increased with the increase of the atomic number of the lanthanide, which corresponds to the results reported in 2021.^[^ ^40^ ^]^

**Figure 5:**
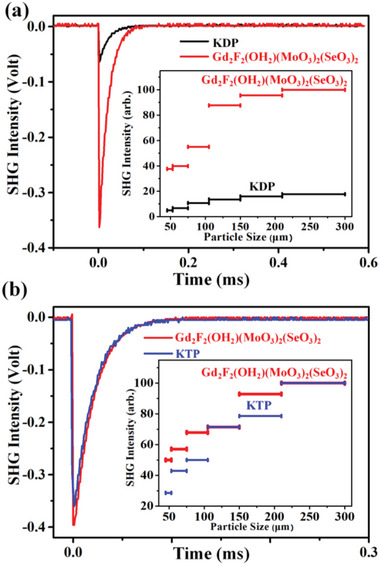
Oscilloscope traces of the SHG signals of Gd_2_F_2_(OH_2_)(MoO_3_)_2_(SeO_3_)_2_ at (a) 1064 nm and (b) 2.05 µm. Plots of SHG intensity versus particle size are shown in the insets. KDP and KTP samples serve as the references at 1064 nm and 2.05 µm, respectively.

To avoid the influence of f‐f transition, the semi‐filled Gd compound was chosen for further research. The SHG intensity on different particle size was studied to evaluate whether it can realize phase matching (**Figure** **5**). From the inset of Figure 5a, we can find that the SHG intensity is increased with the increase of the particle size until it reaches saturation, which indicates that Gd_2_F_2_(OH_2_)(MoO_3_)_2_(SeO_3_)_2_ can realize phase matching at 1064 nm. To explore its performance in near infrared wavelength, we extended the incident wavelength to 2.05 µm. KTP was used as the reference, and its powder SHG intensity is one order of magnitude larger than that of KDP. As Figure 5b shows, the SHG intensity for Gd_2_F_2_(OH_2_)(MoO_3_)_2_(SeO_3_)_2_ is ≈1.2 times that of KTP under the radiation of 2.05 µm laser, which is comparable with that of BiFSeO_3_.^[^ ^41^ ^]^ Furthermore, Gd_2_F_2_(OH_2_)(MoO_3_)_2_(SeO_3_)_2_ can also realize phase matching at 2050 nm. So, Gd_2_F_2_(OH_2_)(MoO_3_)_2_(SeO_3_)_2_ can be used at visible and near‐IR wavelength bands.

**Figure 6:**
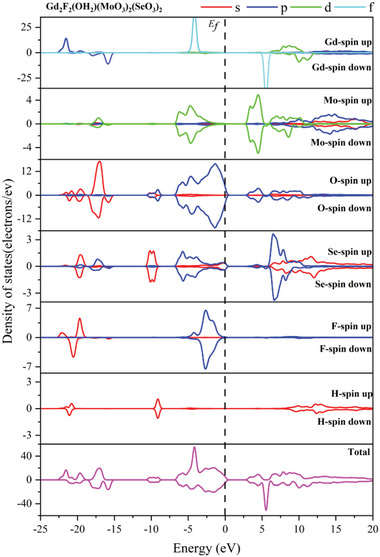
Calculated total and partial density of states of Gd_2_F_2_(OH_2_)(MoO_3_)_2_(SeO_3_)_2_.

The DFT method was employed to investigate the underlying structural and electronic factors contributing to the pronounced SHG effect observed in Gd_2_F_2_(OH_2_)(MoO_3_)_2_(SeO_3_)_2_ (Table S4, Supporting Information). It features an indirect bandgap compound with a calculated bandgap of 1.87 eV (Figure S6, Supporting Information), which is much lower than the experimental result of 3.15 eV. So, a scissor of 1.28 eV was used in the following calculations. As the partial density of states (PDOS) of Gd_2_F_2_(OH_2_)(MoO_3_)_2_(SeO_3_)_2_ shown, Gd demonstrates a significant degree of spin polarization in its states, contrasting with the relatively balanced spin‐up and spin‐down states observed in other atoms (**Figure** **6**). The primary composition of the highest valence band is attributed to the nonbonding states of O‐2p while the conduction band bottom is predominantly governed by the unoccupied Mo‐4d and O‐2p states. So, the band gap of Gd_2_F_2_(OH_2_)(MoO_3_)_2_(SeO_3_)_2_ is determined by Mo and O atoms. Its birefringence was calculated to be 0.143 and 0.133 at 1064 and 2050 nm relatively, which are large enough for the phase‐matching (Figure S7, Supporting Information). The shortest type I phase‐matching SHG wavelength of Gd_2_F_2_(OH_2_)(MoO_3_)_2_(SeO_3_)_2_ was estimated as 420 nm according to the theoretical refractive index dispersion profiles (Figure S8, Supporting Information).

**Figure 7:**
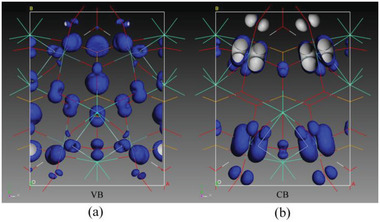
SHG density of *d*_31_ in the valence band (a) and conduction band (b) of Gd_2_F_2_(OH_2_)(MoO_3_)_2_(SeO_3_)_2_.

The largest SHG tensor *d*_31_ was calculated to be 12.97 pmV^−1^, slightly larger than the experimental value of KTP (*d*_33_@1313 nm = 11.1±0.6 pm V^−1^),^[^ ^42^ ^]^ which is consistent with our powder SHG measurements. SHG‐weighed electron density was calculated to represent the SHG effect distributions in Gd_2_F_2_(OH_2_)(MoO_3_)_2_(SeO_3_)_2_. As depicted in **Figure** **7**, the SHG effects in the valence band predominantly arise from the O‐2p nonbonding orbitals, whereas in the conduction band, the SHG process is primarily influenced by the unoccupied electronic states of Mo‐4d in conjunction with O‐2p states. By considering the overall SHG density in both the valence band and conduction band, the respective SHG contribution percentages were calculated as 56.6% for the MoO_6_ octahedra, 24.5% for the SeO_3_ group, and 17.9% for the GdO_7_F_2_ polyhedra. It is evident that the severely distorted MoO_6_ octahedra are the major factor to the SHG effect and each building unit has a beneficial impact on the SHG process, leading to the remarkable properties of Gd_2_F_2_(OH_2_)(MoO_3_)_2_(SeO_3_)_2_ as a promising SHG material.

In addition to the frequency‐doubling property, these compounds may also possess good fluorescence and magnetic properties since they contain active lanthanide ions. Due to the coupling of frequency‐doubling and laser may result self‐frequency‐doubling laser crystals, we focused on their luminescent properties. Except for Gd_2_F_2_(OH_2_)(MoO_3_)_2_(SeO_3_)_2_, which is almost transparent in the range of 2500–800 nm, the fluorescence properties of the other four compounds were explored as follows.

**Figure 8:**
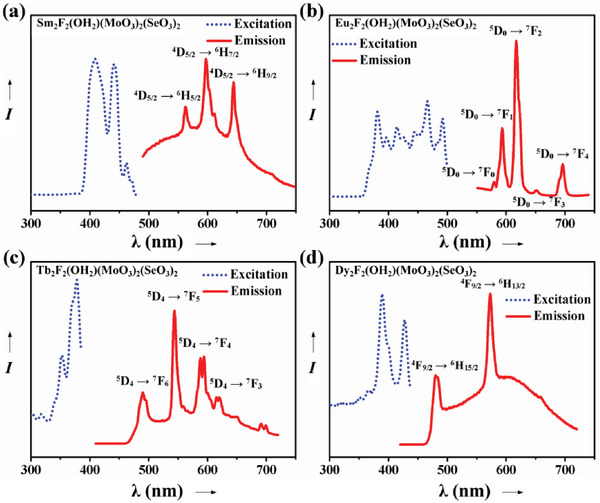
Photoluminescence properties of Ln_2_F_2_(OH_2_)(MoO_3_)_2_(SeO_3_)_2_ [Ln = Sm (a), Eu (b), Tb (c) and Dy (d)].

Sm_2_F_2_(OH_2_)(MoO_3_)_2_(SeO_3_)_2_: As shown in **Figure** **8**a, visible emission spectra have been recorded at 410 nm, 442 nm, and 461 nm under excitation (dotted blue line). Under excitation at 410 nm, the sample emits transitions of ^4^G_5/2_→^6^H_5/2_, ^4^G_5/2_→^6^H_7/2_ and ^4^G_5/2_→^6^H_9/2_ corresponding to the emission of Sm^3+^ at around 562 nm, 597 nm, and 644 nm, respectively (red solid line). This result is consistent with the previously reported for Sm^3+^ ions in the red‐orange range of the spectrum.^[^ ^43^ ^]^

Eu_2_F_2_(OH_2_)(MoO_3_)_2_(SeO_3_)_2_: Eu^3+^ is a well‐known red phosphor^[^ ^44^ ^]^ with a typical strong emission transition ^5^D_0_→^7^F_2_ at ≈612 nm with the red light emitting. The characteristic ^5^D_0_→^7^F_J_ (J = 0, 1, 2, 3, 4) transition peaks of Eu^3+^ exist in compound Eu_2_F_2_(OH_2_)(MoO_3_)_2_(SeO_3_)_2_ with the luminescence range around 580 nm for ^5^D_0_→^7^F_0_, 593 nm for ^5^D_0_→^7^F_1_, 617 nm for ^5^D_0_→^7^F_2_, 651 nm for ^5^D_0_→^7^F_3_ and 696 nm for ^5^D_0_→^7^F_4_ transition (Figure 8b).

Tb_2_F_2_(OH_2_)(MoO_3_)_2_(SeO_3_)_2_: Its known to all, Tb^3+^ is one of the ions producing green light.^[^ ^45^ ^]^ As shown in Figure 8c, Tb_2_F_2_(OH_2_)(MoO_3_)_2_(SeO_3_)_2_ phosphor showed four characteristic Tb^3+^ transitions in the emission spectrum under UV light at 378 nm: ^5^D_4_→^7^F_6_, ^5^D_4_→^7^F_5_, ^5^D_4_→^7^F_4_ and ^5^D_4_→^7^F_3_ at ≈490 nm, 544 nm, 594 nm, and 621 nm, respectively. The ^5^D_4_→^7^F_5_ green emission is the dominant one for this compound.

Dy_2_F_2_(OH_2_)(MoO_3_)_2_(SeO_3_)_2_: Dy^3+^ can give different colors for its luminous behavior.^[^ ^46^ ^]^ Dy^3+^ can be used to obtain green emission (emission peak ≈480 nm), but it is also possible to use Dy^3+^ ions directly to obtain yellow light from a strong emission peak around 574 nm. For Dy_2_F_2_(OH_2_)(MoO_3_)_2_(SeO_3_)_2_ (Figure 8d), under the excitation wavelength of 389 nm, the characteristic strong peaks were found ≈480 and 573 nm, corresponding to ^4^F_9/2_→^6^H_15/2_ and ^4^F_9/2_→^6^H_13/2_ transitions, respectively.

## 3 Conclusion

In summary, the first polar lanthanide d^0^‐TM selenites, namely, Ln_2_F_2_(OH_2_)(MoO_3_)_2_(SeO_3_)_2_ (Ln = Sm, Eu, Gd, Tb and Dy), were achieved by partial fluorination strategy under an acid and HF corrosion resistant supercritical hydrothermal method. Their structures displayed a novel 3D framework consisting of 1D molybdenum selenite chains bridged by Ln_2_F_2_O_12_(OH_2_) dimers. Compounds Sm, Eu, Tb, and Dy can exhibit strong luminescence in orange, red, green, and yellow regions, respectively. Due to the different characteristics of the lanthanide ions, their powder SHG responses showed large differences, ranging from 1.0 to 9.0 × KDP at 1064 nm. Interestingly, the SHG intensity of compound Sm to Tb increases with the increase of the atomic number of the lanthanide element. A detailed study on the SHG property of the half‐filled Gd compound shows Gd_2_F_2_(OH_2_)(MoO_3_)_2_(SeO_3_)_2_ can realize phase matching at both 1064 and 2050 nm and its SHG intensity at 2050 nm can reach to 1.2 × KTP. The strong SHG efficiency was contributed by the synergistic effect of MoO_6_, SeO_3_ and LnO_7_F_2_ groups with percentages of 56.6%, 24.5%, and 17.9%, respectively based on the DFT calculations. This work proved that the corrosion resistant supercritical reactions assisted partial fluorination strategy is an effective method to create multifunctional nonlinear optical materials in lanthanide compounds.

## 4 Experimental Section

All the reagents were obtained from commercial sources and employed without further refinement: Sm_2_O_3_ (Adamas‐beta, 99.9%), Eu_2_O_3_ (Adamas‐beta, 99.9%), Gd_2_O_3_ (Adamas‐beta, 99.9%), Tb_4_O_7_ (Adamas‐beta, 99.9%), Dy_2_O_3_ (Adamas‐beta, 99.9%), MoO_3_ (Adamas‐beta, 99.5%+), hydrofluoric acid (HF, Adamas‐beta, 40%), and SeO_2_ (Adamas‐beta, 99.9%). (***Caution!*** *Hydrofluoric acid is toxic and corrosive! It must be handled with extreme caution and the appropriate protective equipment and training.)*

The single crystals of Ln_2_F_2_(OH_2_)(MoO_3_)_2_(SeO_3_)_2_ (Ln = Sm‐Dy) were synthesized by Ln_2_O_3_ (Ln = Sm, Eu, Gd, and Dy) or Tb_4_O_7_, MoO_3_, SeO_2_ and hydrofluoric acid under supercritical hydrothermal conditions. A mixture of Ln_2_O_3_ (0.349 g, 1 mmol for Sm_2_O_3_, 0.352 g, 1 mmol for Eu_2_O_3_, 0.363 g, 1 mmol for Gd_2_O_3_, 0.374 g, 0.5 mmol for Tb_4_O_7_ and 0.373 g, 1 mmol for Dy_2_O_3_), MoO_3_ (0.288 g, 2.0 mmol), SeO_2_ (0.333 g, 3.0 mmol), hydrofluoric acid (0.5 mL) and 4 mL of H_2_O were added in a graphite tube with cover, which was then placed in a slightly larger quartz tube. Use the flame to groove above the quartz tube to prevent the cover of the graphite tube from spraying out. Finally, the quartz tubes were put in a high‐temperature and high‐pressure reactor with required amount of water. The hydrothermal autoclave was heated to 380 °C and kept at this temperature for 3 days, then cooled down to room temperature at a rate of 6°C h^−1^. The products were filtered with deionized water. Light yellow crystals were obtained with yield of about 20% (based on Ln). Their purities were checked by the powder X‐ray diffraction (PXRD), which were consistent well with the calculated patterns based on the crystal structures (Figure S1, Supporting Information). As shown in the elemental distribution maps, elements of Ln, Mo, Se and F are evenly dispersed in the crystals of Ln_2_F_2_(OH_2_)(MoO_3_)_2_(SeO_3_)_2_ (Ln = Sm‐Dy) (Figure S2, Supporting Information).

Single‐crystal XRD data for the five compounds were collected on an Agilent Technologies SuperNova dual‐wavelength CCD diffractometer with Mo‐Kα radiation (λ = 0.71073 Å) at 293 K. Data reduction was performed with *CrysAlisPro*, and absorption corrections based on the multiscan method were applied.^[^ ^47^ ^]^ The single crystal structures were determined by the direct methods refined by full‐matrix least‐squares fitting on *F^2^* using *SHELXL‐97*.^[^ ^48^ ^]^ All of the atoms were refined with anisotropic thermal parameters and finally converged for *F*_0_^2^ ≥ 2*σ*(*F*_0_^2^). The structural data were also checked for possible missing symmetry with the program *PLATON*, and no higher symmetry was found.^[^ ^49^ ^]^ Crystallographic data and structural refinements of Ln_2_F_2_(OH_2_)(MoO_3_)_2_(SeO_3_)_2_ (Ln = Sm‐Dy) are listed in Table 1. The selected bond distances are listed in Table S1 (Supporting Information).

Powder X‐ray diffraction (PXRD) patterns were collected on a Rigaku MiniFlex II diffractometer using Cu‐Kα radiation in the angular range of 2θ = 5−65° with a step size of 0.02°.

Microprobe elemental analyses and the elemental distribution maps were measured on a field‐emission scanning electron microscope (FESEM, JSM6700F) equipped with an energy‐dispersive X‐ray spectroscope (EDS, Oxford INCA).

FTIR spectra were carried out on a Magna 750 FT‐IR spectrometer using KBr as the diluent in 4000–400 cm^−1^ with a resolution of 2 cm^−1^ at room temperature.

The UV–vis–NIR diffuse reflectance spectra were measured at 200–2500 nm by a PE Lambda 900 UV–vis–NIR spectrophotometer using BaSO_4_ as the reference. Absorption data was calculated from the diffuse reflection data by the Kubelka‐Munk function: α/S = (1‐R)^2^/2R, where α and S represent the absorption coefficient and the scattering coefficient, respectively.^[^ ^50^ ^]^

Thermogravimetric analysis (TGA) was performed on a Netzsch STA 449C instrument with a heating rate of 15 °C min^−1^ under a nitrogen atmosphere from 30 to 1000 °C.

Thermogravimetric‐Mass spectrometer (TG‐MS) analysis was performed on synchronous thermal analyzer (model STA 449 F5) and mass spectrometer (model QMS403C).

Powder SHG measurements were conducted using a modified method of Kurtz and Perry.^[^ ^51^ ^]^ Irradiation laser (λ = 1064 nm and λ = 2.05 µm) is generated by a Nd:YAG solid‐state laser equipped with a Q switch. The Gd_2_F_2_(OH_2_)(MoO_3_)_2_(SeO_3_)_2_ pure crystal samples ground into powder were sieved according to seven different particle size ranges (45−53, 53−75, 75−105, 105−150, 150−210, and 210−300 µm). KH_2_PO_4_ (KDP) and KTiOPO_4_ (KTP) samples in the same size ranges were also prepared, which were used as reference. SHG signals oscilloscope traces of Ln_2_F_2_(OH_2_)(MoO_3_)_2_(SeO_3_)_2_ (Ln = Sm‐Dy) and KDP/KTP samples in the particle size range (150−210 µm) were recorded.

Photoluminescent properties including the emission and excitation spectra in solid state were measured on a FLS920 Edinburgh fluorescence spectrometer.

[Further details of the crystal structure investigation(s) may be obtained from the Fachinformationszentrum Karlsruhe, 76344 Eggenstein‐Leopoldshafen (Germany), on quoting the CCDC depository numbers 2213643−2213647].

## Conflict of Interest

The authors declare no conflict of interest.

## Supporting information

Supporting Information

## Data Availability

The data that support the findings of this study are available from the corresponding author upon reasonable request.
