# Supplementary material for: Ln2F2(OH2)(MoO3)2(SeO3)2: Promising Multifunctional Nonlinear Optical Materials Created by Partial Fluorination Strategy under Corrosion Resistant Supercritical Reactions
Source: Adv Sci (Weinh). 2023 Oct 23;10(34):2304463. doi: 10.1002/advs.202304463 (PMC10700166; doi:10.1002/advs.202304463)
Supplement: Supplementary file 1 — Supporting Information [file ADVS-10-2304463-s001.pdf]

ADVANCED SCIENCE
Open Access

Supporting Information

for *Adv. Sci.*, DOI 10.1002/advs.202304463

$Ln_2F_2(OH_2)(MoO_3)_2(SeO_3)_2$: Promising Multifunctional Nonlinear Optical Materials Created by Partial Fluorination Strategy under Corrosion Resistant Supercritical Reactions

*Yun-Xiang Ma, Peng-Fei Li, Chun-Li Hu, Jiang-Gao Mao and Fang Kong**

Supporting Information

$Ln_2F_2(OH_2)(MoO_3)_2(SeO_3)_2$: Promising Multifunctional Nonlinear Optical Materials Created by Partial Fluorination Strategy under Corrosion Resistant Supercritical Reactions

Yun-Xiang Ma,† Peng-Fei Li,† Chun-Li Hu, Jiang-Gao Mao and Fang Kong*

State Key Laboratory of Structural Chemistry, Fujian Institute of Research on the Structure of Matter, Chinese Academy of Sciences, Fuzhou 350002, PR China

† Y.-X. Ma and P.-F Li contribute equally to this work.

*Corresponding Authors: kongfang@fjirsm.ac.cn

Table of Contents

Section S1. Computational Method……………………………………………………………………S2

Figure S1. PXRD patterns of $Ln_2F_2(OH_2)(MoO_3)_2(SeO_3)_2$ [Ln = Sm (a), Eu (b), Gd (c) Tb (d) and Dy (e)]. S3

Figure S2. SEM images and element mapping of $Ln_2F_2(OH_2)(MoO_3)_2(SeO_3)_2$ [Ln = Sm (a), Eu (b), Gd (c), Tb (d) and Dy (e)]. S4

Figure S3. FTIR spectra of $Ln_2F_2(OH_2)(MoO_3)_2(SeO_3)_2$ [Ln = Sm (a), Eu (b), Gd (c), Tb (d) and Dy (e)]. S5

Figure S4. UV–vis–NIR diffuse reflectance spectra of $Ln_2F_2(OH_2)(MoO_3)_2(SeO_3)_2$ [Ln = Sm (a), Eu (b), Gd (c), Tb (d) and Dy (e)]. S6

Figure S5. TGA curves of $Ln_2F_2(OH_2)(MoO_3)_2(SeO_3)_2$ (Ln = Sm-Dy). S7

Figure S6. Calculated band structures of $Gd_2F_2(OH_2)(MoO_3)_2(SeO_3)_2$. S8

Figure S7. Calculated refractive indices and birefringence of $Gd_2F_2(OH_2)(MoO_3)_2(SeO_3)_2$. S9

Figure S8. Calculated refractive index dispersion curves and the predicted shortest type I phase-matching (PM) SHG wavelength of $Gd_2F_2(OH_2)(MoO_3)_2(SeO_3)_2$. S10

Table S1. Selected important bond lengths [Å] for compounds $Ln_2F_2(OH_2)(MoO_3)_2(SeO_3)_2$ (Ln=Sm-Dy). S11

Table S2. Bond valence sum (BVS) values for compounds $Ln_2F_2(OH_2)(MoO_3)_2(SeO_3)_2$ (Ln=Sm-Dy). S13

Table S3. The thermal stability of some representative selenite SHG materials with halogen ions. S14

Table S4. State energies (eV) of the lowest conduction band (L-CB) and the highest valence band (H-VB) of the $Gd_2F_2(OH_2)(MoO_3)_2(SeO_3)_2$. S15

Reference S16

Section S1. Computational Method

Single-crystal structural data of compounds $Gd_2F_2(OH_2)(MoO_3)_2(SeO_3)_2$ was used for the theoretical calculations. The electronic structures were performed using a plane-wave basis set and pseudo-potentials within density functional theory (DFT) implemented in the total-energy code CASTEP [1]. For the exchange and correlation functional, we chose Perdew–Burke–Ernzerhof (PBE) in the generalized gradient approximation (GGA)[2]. The interactions between the ionic cores and the electrons were described by the ultrasoft pseudopotential[3]. The following valence-electron configurations were considered in the computation: Mo-$4d^55s^1$, Gd-$4f^75s^25p^65d^16s^2$, Se-$4s^24p^4$, O-$2s^22p^4$, F-$2s^22p^5$ and H-$1s^2$. The numbers of plane waves included in the basis sets were determined by cutoff energy of 850 eV for $Gd_2F_2(OH_2)(MoO_3)_2(SeO_3)_2$. The numerical integration of the Brillouin zone were performed using Monkhorst-Pack k-point sampling of 4 × 3 × 3. The other parameters and convergent criteria were the default values of CASTEP code.

The calculations of second-order NLO properties were based on length-gauge formalism within the independent particle approximation [4]. We adopted Chen's static formula, which was derived by Rashkeev *et al.* [5] and later improved by Chen's group. The second-order NLO susceptibility can be expressed as

$$\chi^{\alpha\beta\gamma} = \chi^{\alpha\beta\gamma}(\mathrm{VE}) + \chi^{\alpha\beta\gamma}(\mathrm{VH}) + \chi^{\alpha\beta\gamma}(\text{two bands})$$

where $\chi^{\alpha\beta\gamma}$ (VE) and $\chi^{\alpha\beta\gamma}$ (VH) contribute to $\chi^{\alpha\beta\gamma}$ from virtual-electron processes and virtual-hole processes, respectively, and $\chi^{\alpha\beta\gamma}$ (two bands) contributes to $\chi^{\alpha\beta\gamma}$ from the two-band processes. The formulae for calculating $\chi^{\alpha\beta\gamma}$ (VE), and $\chi^{\alpha\beta\gamma}$ (VH), are given in ref [6].

The calculations of linear optical properties in terms of the complex dielectric function $\varepsilon(\omega) = \varepsilon_1(\omega) + i\varepsilon_2(\omega)$ were made. The imaginary part of the dielectric function ε2 was given in the following equation:

$$\varepsilon^{ij}{}_2(\omega) = \frac{8\pi^2 h^2 e^2}{(m^2 V)} \sum_k \sum_{cv} (f_c - f_v) \frac{p^i_{cv}(k) p^j_{cv}(k)}{E^2_{vc}} \delta\, [E_c(k) - E_v(k) - h\omega]$$

The f_c and f_v represent the Fermi distribution functions of the conduction and valence band. The term $p^i_{cv}(k)$ denotes the momentum matrix element transition from the energy level c of the conduction band to the level v of the valence band at the *k*th point in the Brillouin zone (BZ), and *V* is the volume of the unit cell.

The real part $\varepsilon_1(\omega)$ of the dielectric function $\varepsilon(\omega)$ follows from the Kramer–Kronig relationship. All the other optical constants may be derived from $\varepsilon_1(\omega)$ and $\varepsilon_2(\omega)$. For example, the refractive index $n(\omega)$ can be calculated using the following expression[7]:

$$n(\omega) = \left(\frac{1}{\sqrt{2}}\right)\left[\sqrt{\varepsilon_1^2(\omega) + \varepsilon_2^2(\omega)} + \varepsilon_1(\omega)\right]^{1/2}$$

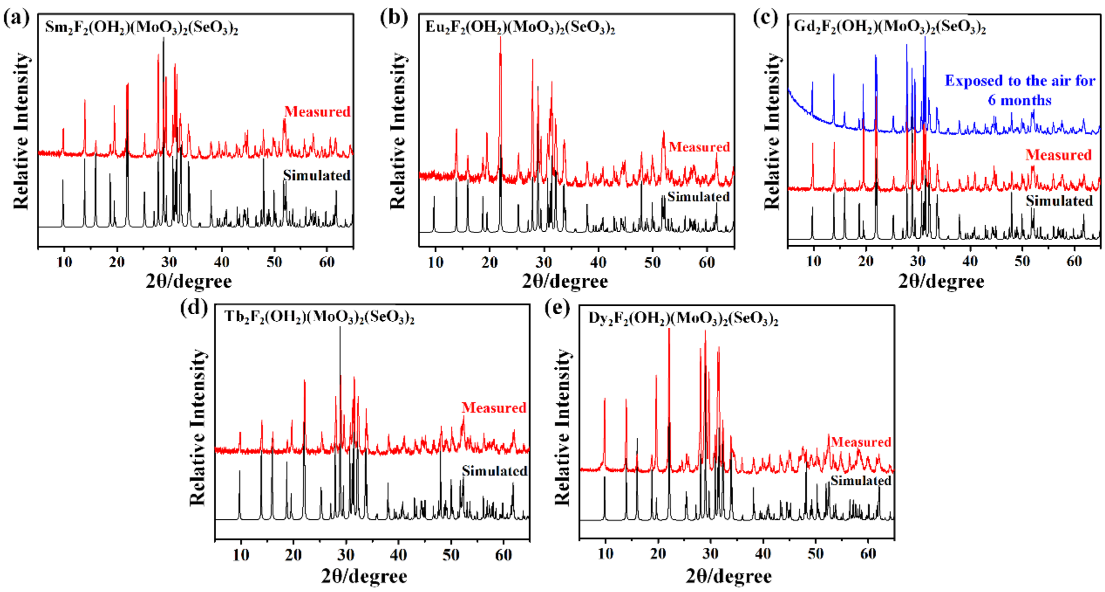

Figure S1. PXRD patterns of $Ln_2F_2(OH_2)(MoO_3)_2(SeO_3)_2$ [Ln = Sm (a), Eu (b), Gd (c) Tb (d) and Dy (e)].

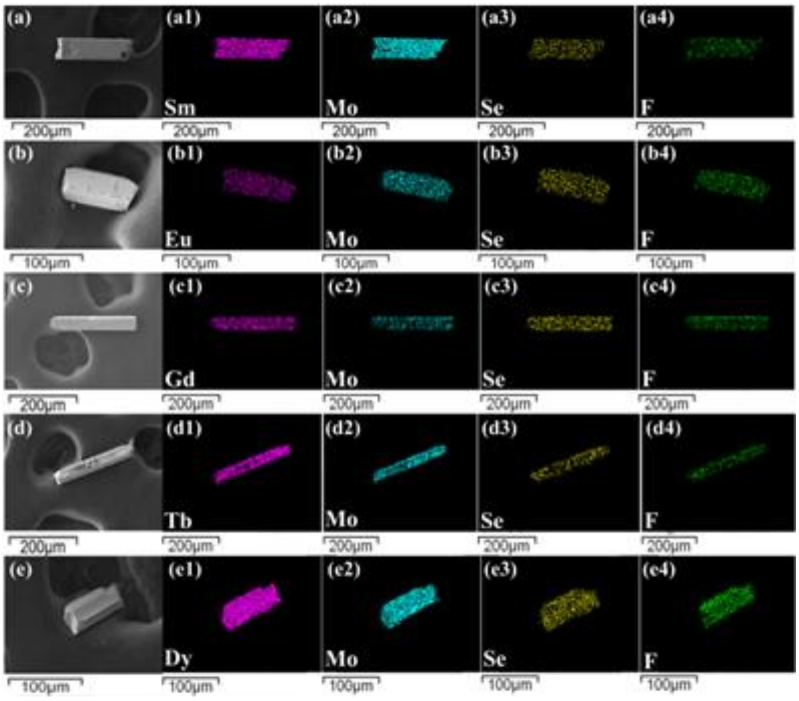

Figure S2. SEM images and element mapping of $Ln_2F_2(OH_2)(MoO_3)_2(SeO_3)_2$ [Ln = Sm (a), Eu (b), Gd (c), Tb (d) and Dy (e)].

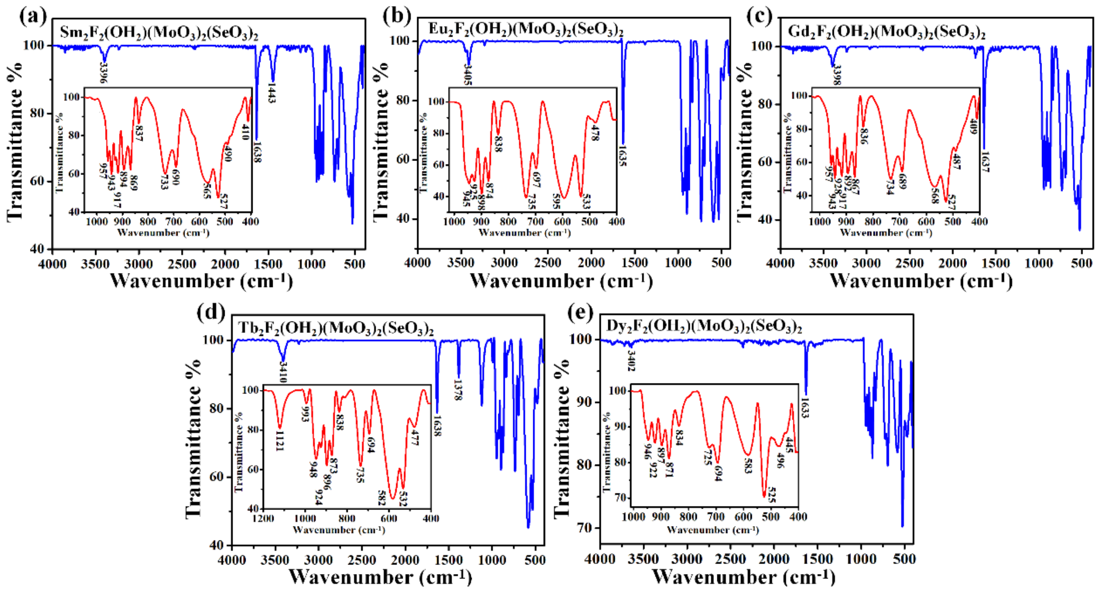

Figure S3. FTIR spectra of $Ln_2F_2(OH_2)(MoO_3)_2(SeO_3)_2$ [Ln = Sm (a), Eu (b), Gd (c), Tb (d) and Dy (e)].

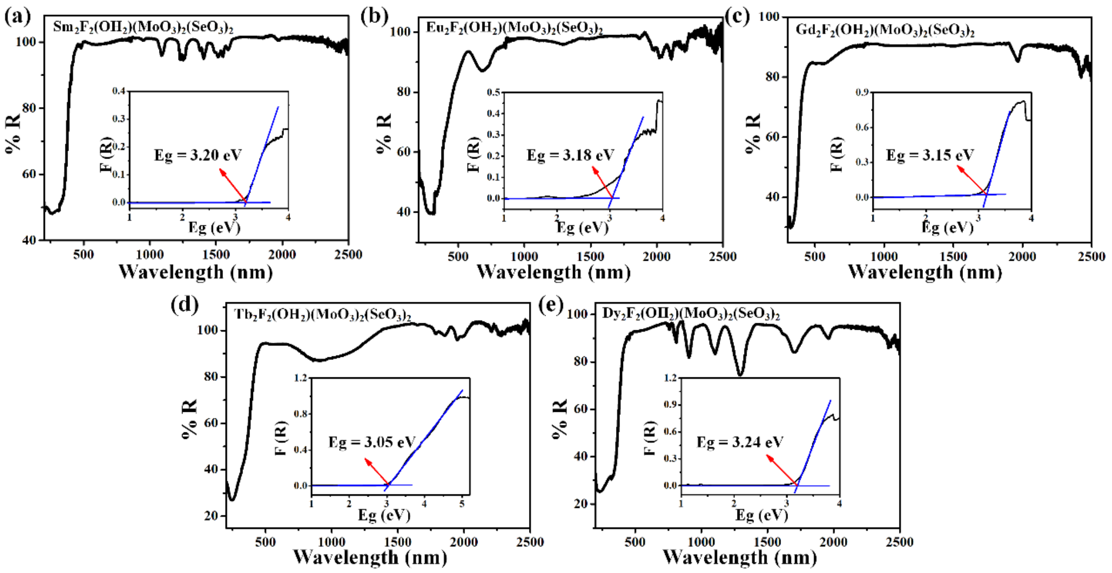

Figure S4. UV–vis–NIR diffuse reflectance spectra of $Ln_2F_2(OH_2)(MoO_3)_2(SeO_3)_2$ [Ln = Sm (a), Eu (b), Gd (c), Tb (d) and Dy (e)].

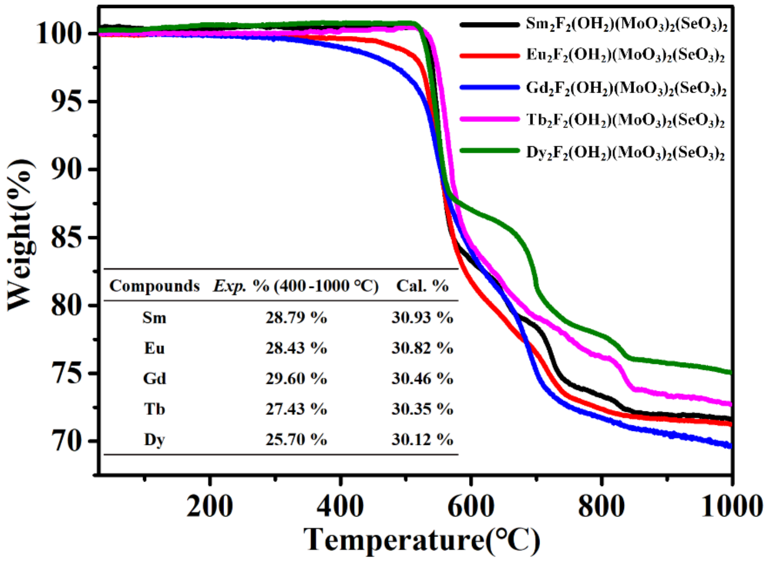

Figure S5. TGA curves of $Ln_2F_2(OH_2)(MoO_3)_2(SeO_3)_2$ (Ln = Sm-Dy).

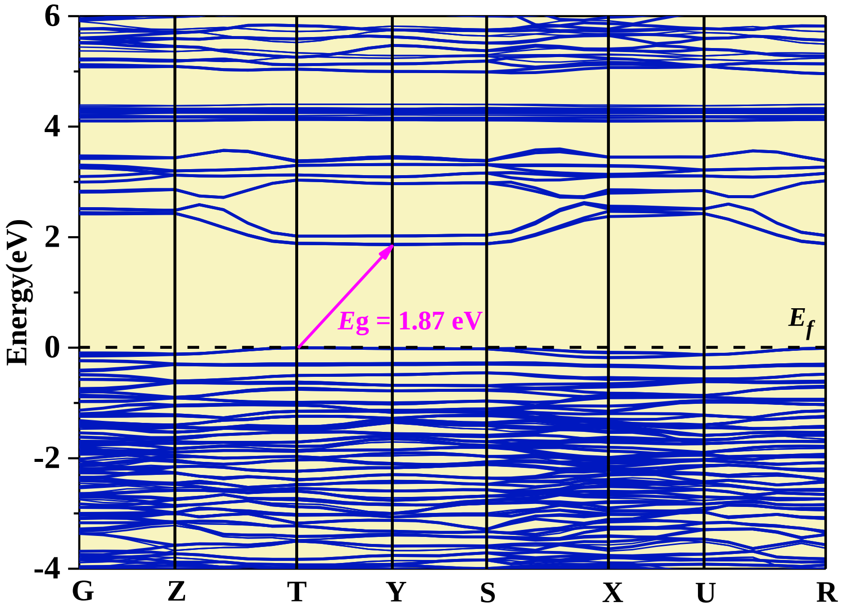

Figure S6. Calculated band structures of $Gd_2F_2(OH_2)(MoO_3)_2(SeO_3)_2$.

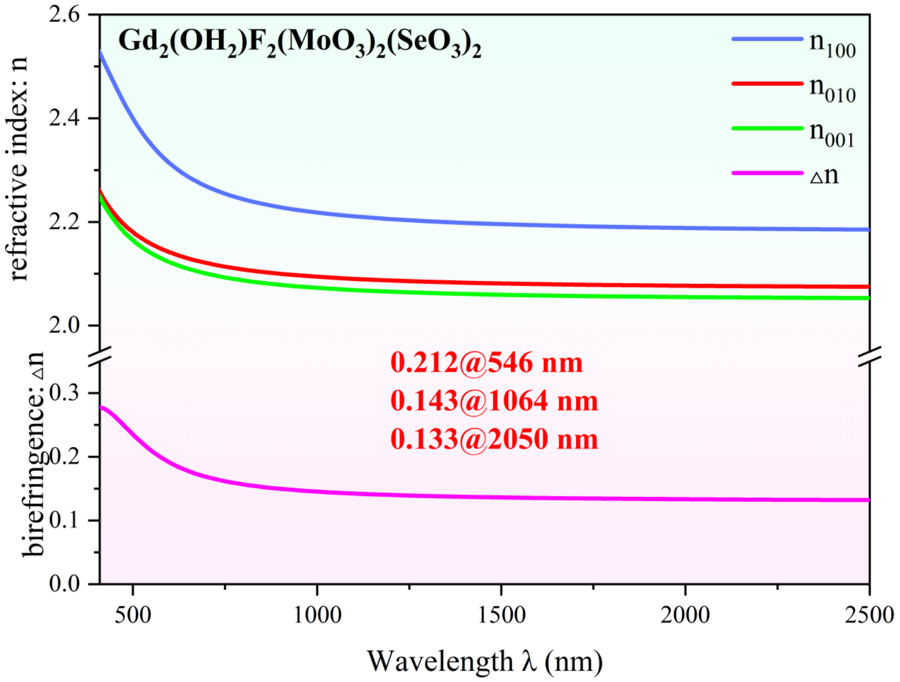

Figure S7. Calculated refractive indices and birefringence of $Gd_2F_2(OH_2)(MoO_3)_2(SeO_3)_2$.

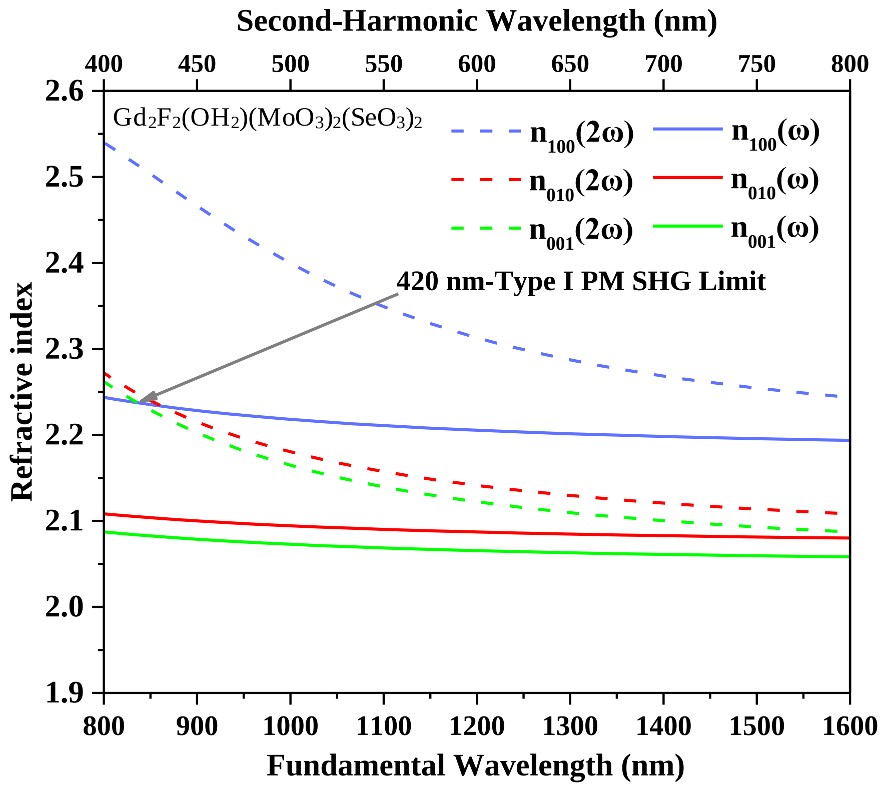

Figure S8. Calculated refractive index dispersion curves and the predicted shortest type I phase-matching (PM) SHG wavelength of $Gd_2F_2(OH_2)(MoO_3)_2(SeO_3)_2$.

Table S1. Selected important bond lengths [Å] for compounds $Ln_2F_2(OH_2)(MoO_3)_2(SeO_3)_2$ (Ln=Sm-Dy).

**$Sm_2F_2(OH_2)$(Mo**		**$Eu_2F_2(OH_2)$(Mo**		**$Gd_2F_2(OH_2)$(Mo**		**$Tb_2F_2(OH_2)$(Mo**		**$Dy_2F_2(OH_2)$(Mo**	
Sm(1)-F	2.340(	Eu(1)-F(	2.321	Gd(1)-F(	2.317	Tb(1)-F	2.307(	Dy(1)-F	2.292
Sm(1)-F	2.340(	Eu(1)-F(	2.321	Gd(1)-F(	2.317	Tb(1)-F	2.307(	Dy(1)-F	2.292
Sm(1)-	2.429(	Eu(1)-O	2.433	Gd(1)-O	2.412	Tb(1)-	2.401(	Dy(1)-O	2.371
Sm(1)-	2.452(	Eu(1)-O	2.433	Gd(1)-O	2.434	Tb(1)-	2.425(	Dy(1)-O	2.406
Sm(1)-	2.452(	Eu(1)-O	2.433	Gd(1)-O	2.434	Tb(1)-	2.425(	Dy(1)-O	2.406
Sm(1)-	2.450(	Eu(1)-O	2.455	Gd(1)-O	2.449	Tb(1)-	2.477(	Dy(1)-O	2.443
Sm(1)-	2.450(	Eu(1)-O	2.455	Gd(1)-O	2.449	Tb(1)-	2.477(	Dy(1)-O	2.443
Sm(1)-	2.508(	Eu(1)-O	2.499	Gd(1)-O	2.451	Tb(1)-	2.419(	Dy(1)-O	2.430
Sm(1)-(	2.519(	Eu(1)-(	2.487	Gd(1)-(	2.465	Tb(1)-(	2.475(	Dy(1)-(	2.438
Sm(2)-F	2.353(	Eu(2)-F(	2.340	Gd(2)-F(	2.336	Tb(2)-F	2.309(	Dy(2)-F	2.290
Sm(2)-F	2.353(	Eu(2)-F(	2.340	Gd(2)-F(	2.336	Tb(2)-F	2.309(	Dy(2)-F	2.290
Sm(2)-	2.354(	Eu(2)-O	2.354	Gd(2)-O	2.368	Tb(2)-	2.309(	Dy(2)-O	2.318
Sm(2)-	2.461(	Eu(2)-O	2.447	Gd(2)-O	2.417	Tb(2)-	2.433(	Dy(2)-O	2.424
Sm(2)-	2.461(	Eu(2)-O	2.447	Gd(2)-O	2.417	Tb(2)-	2.433(	Dy(2)-O	2.424
Sm(2)-	2.496(	Eu(2)-O	2.504	Gd(2)-O	2.486	Tb(2)-	2.474(	Dy(2)-O	2.472
Sm(2)-	2.496(	Eu(2)-O	2.504	Gd(2)-O	2.486	Tb(2)-	2.474(	Dy(2)-O	2.472
Sm(2)-	2.519(	Eu(2)-O	2.502	Gd(2)-O	2.511	Tb(2)-	2.470(	Dy(2)-O	2.462
Sm(2)-	2.521(	Eu(2)-O	2.513	Gd(2)-O	2.504	Tb(2)-	2.473(	Dy(2)-O	2.456
Se(1)-O	1.654(	Se(1)-O(	1.651	Se(1)-O(	1.642	Se(1)-	1.656(	Se(1)-O	1.637
Se(1)-O	1.738(	Se(1)-O(	1.741	Se(1)-O(	1.756	Se(1)-	1.738(	Se(1)-O	1.742
Se(1)-O	1.738(	Se(1)-O(	1.741	Se(1)-O(	1.756	Se(1)-	1.738(	Se(1)-O	1.742
Se(2)-O	1.676(	Se(2)-O(	1.681	Se(2)-O(	1.678	Se(2)-	1.689(	Se(2)-O	1.699
Se(2)-O	1.708(	Se(2)-O(	1.716	Se(2)-O(	1.745	Se(2)-	1.721(	Se(2)-O	1.715
Se(2)-O	1.708(	Se(2)-O(	1.716	Se(2)-O(	1.745	Se(2)-	1.721(	Se(2)-O	1.715
Mo(1)-	1.732(	Mo(1)-	1.714	Mo(1)-O	1.715	Mo(1)-	1.731(	Mo(1)-	1.714
Mo(1)-	1.727(	Mo(1)-	1.735	Mo(1)-O	1.725	Mo(1)-	1.723(	Mo(1)-	1.722
Mo(1)-	1.922(	Mo(1)-	1.924	Mo(1)-O	1.937	Mo(1)-	1.925(	Mo(1)-	1.926
Mo(1)-	1.939(	Mo(1)-	1.944	Mo(1)-O	1.942	Mo(1)-	1.946(	Mo(1)-	1.944
Mo(1)-	2.198(	Mo(1)-	2.189	Mo(1)-O	2.180	Mo(1)-	2.212(	Mo(1)-	2.180
Mo(1)-	2.227(	Mo(1)-	2.229	Mo(1)-O	2.219	Mo(1)-	2.223(	Mo(1)-	2.218

Symmetry transformations used to generate equivalent atoms: #1 -x+2, y, z; #2 -x+3/2, -y+1, z-1/2; #3 x, y-1, z; #4 -x+2, y-1, z; #5 x+1/2, -y+1, z-1/2; #6 x+1/2, -y+1, z+1/2; #7 -x+3/2, -y+1, z+1/2;

#8 -x+1, y, z.

Table S2. Bond valence sum (BVS) values for compounds $Ln_2F_2(OH_2)(MoO_3)_2(SeO_3)_2$ (Ln=Sm-Dy).

**$Sm_2F_2(OH_2)$(Mo**		**$Eu_2F_2(OH_2)$(Mo**		**$Gd_2F_2(OH_2)$(Mo**		**$Tb_2F_2(OH_2)$(Mo**		**$Dy_2F_2(OH_2)$(Mo**	
Eleme	BVS	Eleme	BVS	Eleme	BVS	Elemen	BVS	Elemen	BVS
Sm1	3.28	Eu1	3.27	Gd1	3.27	Tb1	3.22	Dy1	3.24
Sm2	3.23	Eu2	3.18	Gd2	3.16	Tb2	3.21	Dy2	3.16
Mo1	5.99	Mo1	6.02	Mo1	6.06	Mo1	5.97	Mo1	6.10
Se1	3.96	Se1	3.96	Se1	3.90	Se1	3.96	Se1	4.01
Se2	4.08	Se2	4.01	Se2	3.82	Se2	3.94	Se2	3.95
O1W	-2.48	O1W	-2.50	O1W	-2.52	O1W	-2.50	O1W	-2.55
F1	-0.74	F1	-0.74	F1	-0.72	F1	-0.73	F1	-0.74
O1	-2.31	O1	-2.29	O1	-1.99	O1	-1.97	O1	-2.02
O2	-2.02	O2	-2.01	O2	-2.02	O2	-2.02	O2	-2.07
O3	-2.15	O3	-2.11	O3	-2.12	O3	-2.10	O3	-2.07
O4	-2.11	O4	-2.08	O4	-2.01	O4	-2.04	O4	-2.08
O5	-1.94	O5	-2.00	O5	-2.00	O5	-1.93	O5	-1.99
O6	-2.00	O6	-1.97	O6	-2.00	O6	-2.01	O6	-2.02
O7	-2.24	O7	-2.23	O7	-2.20	O7	-2.27	O7	-2.24
O8	-2.14	O8	-2.12	O8	-2.12	O8	-2.12	O8	-2.13

Table S3. The thermal stability of some representative selenite SHG materials with halogen ions.

Materials	Thermal stability	SHG intensity	Ref.
$Na_3Ti_3O_3(SeO_3)_4F$	430 ℃	6 × KDP	8
$Pb_2GaF_2(SeO_3)_2Cl$	425 ℃	4.5 × KDP	9
$Cs(TiOF)_3(SeO_3)_2$	420 ℃	5 × KDP	10
**$Gd_2F_2(OH_2)(MoO_3)_2(SeO_3)_2$**	**400 ℃**	**5.7 ×KDP**	**This Work**
$Pb_2TiOF(SeO_3)_2Cl$	389 ℃	9.6 × KDP	11
$PbCdF(SeO_3)(NO_3)$	385 ℃	2.6 × KDP	12
$RbGa_3F_6(SeO_3)_2$	370 ℃	5.6 × KDP	13
$CsGa_3F_6(SeO_3)_2$	370 ℃	5.4 × KDP	13
$Pb_2NbO_2(SeO_3)_2Cl$	365 ℃	2.3 × KDP	11
$PbBi(SeO_3)_2F$	330 ℃	10.5 × KDP	14
$Pb_2Bi(SeO_3)_2Cl_3$	330 ℃	13.5 × KDP	14
$BiFSeO_3$	300 ℃	13.5 × KDP	15
$Pb_2(V_2O_4F)(VO_2)(SeO_3)_3$	280 ℃	0.3 × KDP	16
$Ba(MoO_2F)_2(SeO_3)_2$	250 ℃	2.8 × KDP	17

Table S4. State energies (eV) of the lowest conduction band (L-CB) and the highest valence band (H-VB) of the $Gd_2F_2(OH_2)(MoO_3)_2(SeO_3)_2$.

Compound	k-point	L-CB	H-VB
$Gd_2F_2(OH_2)(MoO_3)_2(SeO_3)_2$	G (0.000, 0.000, 0.000)	2.419813	-0.10204
	Z (0.000, 0.000, 0.500)	2.431896	-0.12259
	T (-0.500, 0.000, 0.500)	1.886110	0
	Y (-0.500, 0.000, 0.000)	1.865648	-0.01329
	S (-0.500, 0.500, 0.000)	1.880375	-0.02010
	X (0.000, 0.500, 0.000)	2.374111	-0.09375
	U (0.000, 0.500, 0.500)	2.426110	-0.12842
	R (-0.500, 0.500, 0.500)	1.879983	-0.00394

Reference

1. Segall, M. D.; Lindan, P. J. D.; Probert, M. J.; Pickard, C. J.; Hasnip, P. J.; Clark, S. J.; Payne, M. C., First-principles simulation: ideas, illustrations and the CASTEP code. *Phys-Condens Mat.* **2002,** *14* (11), 2717-2744.
2. Milman, V.; Winkler, B.; White, J. A.; Pickard, C. J.; Payne, M. C.; Akhmatskaya, E. V.; Nobes, R. H., Electronic structure, properties, and phase stability of inorganic crystals: A pseudopotential plane-wave study. *Int. J. Quantum. Chem.* **2000,** *77* (5), 895-910.
3. Perdew, J. P.; Burke, K.; Ernzerhof, M., Generalized Gradient Approximation Made Simple. *Phys. Rev. Lett.* **1996,** *77* (18), 3865-3868.
4. Aversa, C.; Sipe, J. E., Nonlinear optical susceptibilities of semiconductors: Results with a length-gauge analysis. *Phys. Rev. B: Condens. Matter Mater. Phys.* **1995,** *52* (20), 14636-14645.
5. Rashkeev, S. N.; Lambrecht, W. R. L.; Segall, B., Efficientab initiomethod for the calculation of frequency-dependent second-order optical response in semiconductors. *Phys. Rev. B: Condens. Matter Mater. Phys.* **1998,** *57* (7), 3905-3919.
6. Lin, J.; Lee, M.-H.; Liu, Z.-P.; Chen, C.; Pickard, C. J., Mechanism for linear and nonlinear optical effects inβ−BaB2O4crystals. *Phys. Rev. B: Condens. Matter Mater. Phys.* **1999,** *60* (19), 13380-13389.
7. Vanderbilt, D., Soft self-consistent pseudopotentials in a generalized eigenvalue formalism. *Phys Rev B Condens Matter.* **1990,** *41* (11), 7892-7895.
8. Yan, S. N.; Wang, X. X.; Hu, C. L.; Li, B. X.; Kong, F.; Mao, J. G., $Na_3Ti_3O_3(SeO_3)_4F$: A Phase-Matchable Nonlinear-Optical Crystal with Enlarged Second-Harmonic-Generation Intensity and Band Gap. *Inorg. Chem.* **2022,** *61* (5), 2686-2694.
9. You, F. G.; Liang, F.; Huang, Q.; Hu, Z. G.; Wu, Y. C.; Lin, Z. S., $Pb_2GaF_2(SeO_3)_2Cl$: Band Engineering Strategy by Aliovalent Substitution for Enlarging Bandgap while Keeping Strong Second Harmonic Generation Response. *J. Am. Chem. Soc.* **2019,** *141* (2), 748-752.

10. Cao, X. L.; Hu, C. L.; Kong, F.; Mao, J. G., $Cs(TaO_2)_3(SeO_3)_2$ and $Cs(TiOF)_3(SeO_3)_2$: structural and second harmonic generation changes induced by the different d^0-TM coordination octahedra. *Inorg. Chem.* **2015,** *54* (8), 3875-3882.

11. Cao, X. L.; Hu, C. L.; Xu, X.; Kong, F.; Mao, J. G., $Pb_2TiOF(SeO_3)_2Cl$ and $Pb_2NbO_2(SeO_3)_2Cl$: small changes in structure induced a very large SHG enhancement. *Chem. Commun.* **2013,** *49* (85), 9965-9967.

12. Ma, Y. X.; Hu, C. L.; Li, B. X.; Kong, F.; Mao, J. G., $PbCdF(SeO_3)(NO_3)$: A Nonlinear Optical Material Produced by Synergistic Effect of Four Functional Units. *Inorg. Chem.* **2018,** *57* (18), 11839-11846.

13. Wu, C.; Jiang, X. X.; Lin, L.; Lin, Z. S.; Huang, Z. P.; Humphrey, M. G.; Zhang, C., $AGa_3F_6(SeO_3)_2$ (A = Rb, Cs): A New Type of Phase-Matchable Hexagonal Tungsten Oxide Material with Strong Second-Harmonic Generation Responses. *Chem. Mater.* **2020,** *32* (16), 6906-6915.

14. Jia, Y. J.; Zhang, X. Y.; Chen, Y. G.; Jiang, X. X.; Song, J. N.; Lin, Z. S.; Zhang, X. M., $PbBi(SeO_3)_2F$ and $Pb_2Bi(SeO_3)_2Cl_3$: Coexistence of Three Kinds of Stereochemically Active Lone-Pair Cations Exhibiting Excellent Nonlinear Optical Properties. *Inorg. Chem.* **2022,** *61* (39), 15368-15376.

15. Liang, M. L.; Hu, C. L.; Kong, F.; Mao, J. G., $BiFSeO_3$: An Excellent SHG Material Designed by Aliovalent Substitution. *J. Am. Chem. Soc.* **2016,** *138* (30), 9433-9436.

16. Lin, L.; Jiang, X. X.; Wu, C.; Lin, Z. S.; Huang, Z. P.; Humphrey, M. G.; Zhang, C., First chiral fluorinated lead vanadate selenite $Pb_2(V_2O_4F)(VO_2)(SeO_3)_3$ with five asymmetric motifs and large optical properties. *Dalton Trans.* **2021,** *50* (21), 7238-7245.

17. Liang, M. L.; Ma, Y. X.; Hu, C. L.; Kong, F.; Mao, J. G., $Ba(MoO_2F)_2(QO_3)_2$ (Q = Se, Te): Partial Fluorination of MoO_6 Octahedra Enabling Two Polar Solids with Strong and Phase Matchable SHG Response. *Chem. Mater.* **2020,** *32* (22), 9688-9695.
